# Supplementary material for: Spike deep mutational scanning helps predict success of SARS-CoV-2 clades
Source: Nature. 2024 Jul 3;631(8021):617–26. doi: 10.1038/s41586-024-07636-1 (PMC11254757; doi:10.1038/s41586-024-07636-1)
Supplement: Supplementary file 1 — Supplementary Methods, Figs. 1–5, Table 1 and referencs. [file 41586_2024_7636_MOESM1_ESM.pdf]

nature portfolio

https://doi.org/10.1038/s41586-024-07636-1

Supplementary information

Spike deep mutational scanning helps predict success of SARS-CoV-2 clades

In the format provided by the authors and unedited

Full-spike deep mutational scanning helps predict the evolutionary success of SARS-CoV-2 clades

Bernadeta Dadonaite[1], Jack Brown[2], Teagan E McMahon[1], Ariana G Farrell[1], Marlin D Figgins[3,4], Daniel Asarnow[2], Cameron Stewart[2], Jimin Lee[2], Jenni Logue[5], Trevor Bedford[3,6,8], Ben Murrell[7], Helen Y. Chu[5], David Veesler[2,8], Jesse D Bloom[1,8,#]

[1] Basic Sciences Division and Computational Biology Program, Fred Hutchinson Cancer Center, Seattle, Washington, 98109, USA
[2] Department of Biochemistry, University of Washington, Seattle, Washington, USA
[3] Vaccine and Infectious Disease Division, Fred Hutchinson Cancer Center, Seattle, WA, USA
[4] Department of Applied Mathematics, University of Washington, Seattle, WA, USA
[5] University of Washington, Department of Medicine, Division of Allergy and Infectious Diseases, Seattle, WA
[6] Department of Epidemiology, University of Washington, Seattle, WA, USA
[7] Department of Microbiology, Tumor and Cell Biology, Karolinska Institutet, Stockholm, Sweden
[8] Howard Hughes Medical Institute, Seattle, WA, 98195, USA

[#] Lead Contact jbloom@fredhutch.org

Supplementary Methods 1
- Design of deep mutational scanning libraries 1
- Production of plasmid libraries used to generate deep mutational scanning libraries 1
- Production of cell stored deep mutational scanning libraries 2
- Cell entry effect measurement using deep mutational scanning libraries 3
- Use of non-neutralizable standard for ACE2 binding and serum selection experiments 3
- Recombinant Protein Production 4
- ACE2 binding measurement using deep mutational scanning libraries 4
- Mass photometry 5
- Serum escape mapping using deep mutational scanning libraries 5
- Validation of escape using standard pseudovirus neutralization assay 6
- Cells 6
- Ethics statement 6
- Comparison of deep mutational scanning phenotypes to changes in clade growth 7

Supplementary table 1: Information on sera used in this study 9
Supplementary figure 1: BA.2 and its mutant spike preparations 10
Supplementary figure 2: Mass photometry measurements for individual BA.2 spike variants 12
Supplementary figure 3: Mass photometry measurements for individual XBB.1.5 spike variants 13
Supplementary figure 4: Ability of various spike properties to distinguish the actual BA.2.86 and BA.2.86-descended clades from randomly mutated sequences 15
Supplementary figure 5: Example of model fit to lineage counts 17
Supplementary references 18

Supplementary Methods

Design of deep mutational scanning libraries

Deep mutational scanning libraries were designed with codon-optimized XBB.1.5 and BA.2 spikes. The sequence of the XBB.1.5 spike is at https://github.com/jbloomlab/SARS-CoV-2-XBB.1.5_Spike_DMS_validations/blob/main/plasmid_maps/3809_pH2rU3_ForInd_XBB15_Sinobiological_CMV_ZsGT2APurR.gb and the BA.2 spike is at https://github.com/jbloomlab/SARS-CoV-2-XBB.1.5_Spike_DMS_validations/blob/main/plasmid_maps/3332_pH2rU3_ForInd_Omicron_sinobiological_BA2_B11529_Spiked21_T7_CMV_ZsGT2APurR.gb. Note that due to an error on our part early in library design, the XBB.1.5 spike used for libraries lacks F490S mutation present in XBB* variants.

The XBB.1.5 full spike libraries were designed to include all accessible and tolerated mutations by including mutations that appeared in more than 50 sequences on GISAID[76], occurred independently at least 15 times on pre-built SARS-CoV-2 phylogenies from UShER[77] or occurred independently at least 2 times in any of the following clades: BA.2.75, BQ.1.1, XBB, XBB.1.5. Deletions that met the above criteria were only included if they occurred in the NTD and we specifically added deletions at sites 342-349, 444-449, and 483-486. We also performed saturating mutagenesis on the sites that met the following criteria: occurred independently at least 2500 times on pre-built SARS-CoV-2 phylogenies from UShER or occurred independently at least 100 times in the clades mentioned above. We also saturated mutations at sites that had strong antigenic effects or otherwise were of special interest[1,8] full list of these sites can be found at https://github.com/dms-vep/SARS-CoV-2_XBB.1.5_spike_DMS/blob/main/library_design/config.yaml under *sites_to_saturate*. The full list of mutations included in the XBB.1.5 full spike libraries can be found at https://github.com/dms-vep/SARS-CoV-2_XBB.1.5_spike_DMS/blob/main/library_design/results/mutation_design_classification.csv. As shown in Figure 1b this design strategy biases libraries to contain mostly functional mutations. The reason for choosing such a strategy is: (i) it makes variants with multiple mutations more likely to remain functional and (i) it limits the number of mutations that need to be included in the final library.

For the XBB.1.5 RBD-only libraries, every position in the RBD (positions 331-531) was mutagenized to all possible amino acids.

For the BA.2 full spike libraries the design of mutations to be included in the library was performed the same way as described previously for BA.1 libraries[4]. The final list of mutations in BA.2 libraries can be found at https://github.com/dms-vep/SARS-CoV-2_Omicron_BA.2_spike_DMS/blob/main/library_design/results/aggregated_mutations.csv. Note that the BA.2 libraries used in this study are the same ones briefly described in Haddox at al. 2023[10].

Analysis pipelines for designing mutagenesis primers are provided at https://github.com/dms-vep/SARS-CoV-2_XBB.1.5_spike_DMS/tree/main/library_design for XBB.1.5 full spike libraries, at https://github.com/dms-vep/SARS-CoV-2_XBB.1.5_RBD_DMS/tree/main/library_design for XBB.1.5 RBD-only libraries, and at https://github.com/dms-vep/SARS-CoV-2_Omicron_BA.2_spike_DMS/tree/main/library_design for BA.2 libraries.

Production of plasmid libraries used to generate deep mutational scanning libraries

Libraries of lentivirus backbone plasmids containing mutagenised XBB.1.5 or BA.2 spikes were made as described previously[5]. In brief, primers containing desired mutations described above were ordered from IDT as Oligo Pools. Full list of these primers for XBB.1.5 full spike library can be found at https://github.com/dms-vep/SARS-CoV-2_XBB.1.5_spike_DMS/blob/main/library_design/results/oPools.csv , for for XBB.1.5 RBD only library at https://github.com/dms-vep/SARS-CoV-2_XBB.1.5_RBD_DMS/blob/main/library_design/results/oPools.csv , and for BA.2 library at https://github.com/dms-vep/SARS-CoV-2_Omicron_BA.2_spike_ACE2_binding/tree/main/library_design/results (see csv files ending in *oPool.csv*). These primers were used to mutagenize spike sequences using PCR that involves multiple rounds of PCR mutagenesis reactions[84]. Number of PCR rounds and cycles determines the number of mutations per spike introduced and we targeted ~2-3 mutations per spike, although the precise number of mutations per spike is determined only after lentiviral genomes have been integrated into cells and sequenced with long-read sequencing (see *Long-read PacBio sequencing for variant-barcode linkage* section below). For both XBB.1.5 full spike and RBD-only

libraries we pooled spike mutagenesis primers at 2:1 molar ratio between mutations that occur independently multiple times on spike phylogenetic tree and those that occurred multiple times on spike sequences deposited on GISAID database (for RBD only libraries the latter included all possible RBD mutations). For both XBB.1.5 full spike and RBD-only libraries a single round of 10 PCR cycles was used to mutagenize the spike sequence. For BA.2 full spike libraries the same primer pooling strategy and the same number of mutagenesis cycles were used as described for BA.1 libraries[4]. Template spike sequences used for mutagenesis were amplified from https://github.com/jbloomlab/SARS-CoV-2-XBB.1.5_Spike_DMS_validations/blob/main/plasmid_maps/3809_pH2rU3_ForInd_XBB15_Sinobiological_CMV_ZsGT2APurR.gb plasmid for XBB.1.5 libraries and from https://github.com/jbloomlab/SARS-CoV-2-XBB.1.5_Spike_DMS_validations/blob/main/plasmid_maps/3332_pH2rU3_ForInd_Omicron_sinobiological_BA2_B11529_Spiked21_T7_CMV_ZsGT2APurR.gb plasmid for BA.2 libraries. Spikes for both variants were amplified using *VEP_amp_for* (5′CAGCCGAGCCACATCGCTC) and 3′rev_lib_LinJoin_KHDC (5′CGGAAGAGCGTCGTGTAGGGAAAG) primers. After mutagenesis reaction spike sequences were barcoded in a PCR reaction using primers that contained a unique 16 nucleotide barcode that adds barcodes downstream of spike STOP codon. All libraries had two biological replicates (Lib-1 and Lib-2), which represent two independently produced libraries where mutations in spike are associated with unique barcodes in unique combinations with other mutations. Mutagenised and barcoded spike sequence templates were then added into MluI and XbaI digested lentivirus backbone (Addgene #204579) using HiFi reaction (NEB E2621L). Ampure XP bead purified HiFi reactions were then electroporated into 10-beta electrocompetent E. coli cells (NEB, C3020K) and plated overnight. At least 10 electroporation reactions were performed for each plasmid library in order to produce > 2 million CFUs per library. High diversity of barcoded genomes is required in the later steps of library production in order to minimize barcode duplication, which may happen during lentivirus recombination. For each library bacterial colonies were scraped from overnight plates, pooled and QIAGEN HiSpeed Plasmid Maxi Kit (Cat. No. 12662) was used to prepare plasmid pools used for virus library production.

Production of cell stored deep mutational scanning libraries

Steps for producing cell-stored spike deep mutational scanning libraries have been described in detail previously[4] . In brief, two 6-well plates of 293T cells were transfected with plasmid pools described above, lentivirus helper plasmids (BEI: NR-52517, NR-52519, NR-52518) and VSV-G expression plasmid (Addgene #204156). This produced a VSV-G pseudotyped lentivirus pool carrying mutagenised spike sequences in their genomes. VSV-G pseudotyped viruses were then used to infect 293T-rtTA cells at low multiplicity of infection so that no more than one virus would infect each cell. Reverse tetracycline-controlled transactivator (rtTA) is required to induce expression from inducible TRE3G promoter in the lentivirus backbone in the presence of doxycycline (see Addgene #204579 plasmid structure). Note, that 293T-rtTA cells used here is a specific cell clone we isolated when producing rtTA overexpressing cells, which is especially good at producing high titers virus stocks that are required for successful library production. We described production of these 293T-rtTA cells previously[4]. VSV-G infection step was also used to bottleneck the libraries to the desired number of variants; we aimed for between 50,000 and 100,000 variants per library. Final number of variants in each library is shown in **Extended Data Fig. 1a**. After VSV-G infection, cells with successful lentivirus integration were selected for using puromycin. Puromycin selection was performed until visual inspection showed a pure population of cells express zsGreen (which is part of lentivirus backbone, see plasmid Addgene #204579). At this point all cell stored libraries were frozen until further use.

Long-read PacBio sequencing for variant-barcode linkage

Analysis of linkage between mutations in lentivirus backbone encoded spikes and the barcodes they are associated with was performed using long read PacBio sequencing as described previously[4]. First, we rescued VSV-G pseudotyped viruses from cell-stored libraries by transfecting those cells with lentivirus helper and VSV-G expression plasmids. VSV-G pseudotyped viruses produced from these libraries were then used to infect 293T cells and nonintegrated viral genomes were recovered as described previously[4]. To avoid strand switching and mixing of variant-barcode pairs viral genomes were then minimally PCR amplified using primers with tags that allow to detect strand switching via sequencing. Long read sequencing was performed with PacBio Sequel IIe machine. Consensus variant-barcode sequence was determined requiring at least two CCS sequences per barcode. Variant-barcode lookup tables for each library can be found at:

- For XBB.1.5 full spike library https://github.com/dms-vep/SARS-CoV-2_XBB.1.5_spike_DMS/blob/main/results/variants/codon_variants.csv
- For XBB.1.5 RBD only https://github.com/dms-vep/SARS-CoV-2_XBB.1.5_RBD_DMS/blob/main/results/variants/codon_variants.csv
- For BA.2 library https://github.com/dms-vep/SARS-CoV-2_Omicron_BA.2_spike_ACE2_binding/blob/main/results/variants/codon_variants.csv

Long read sequencing data was also used to determine the average spike mutation frequency in each library. For XBB.1.5 full spike library Lib-1 and Lib-2 mutation frequency 1.91 mutations per spike. For XBB.1.5 RBD only library Lib-1 had an average of 1.82 mutations per spike and Lib-2 had an average of 1.9 mutations per spike. BA.2 libraries had an average of 2.32 and 2.33 mutations per spike for Lib-1 and Lib-2 libraries, respectively.

Cell entry effect measurement using deep mutational scanning libraries

Cell entry effects for each variant were measured as described previously[4]. In brief, ~1.5 million transcription units of spike pseudotyped library viruses and ~5 million of VSV-G pseudotyped transcription units made from the same cell-stored libraries were used to infect target cells. For spike pseudotyped libraries 293T-cells either overexpressing high amounts of ACE2 (described in Crawford et al 2020[5]) or cell expressing medium amount of ACE2 (described in Farrell et al. 2021[32]) were used. Whenever cells were plated for infection with spike-pseudotyped viruses (including for ACE2 and sera selections described below) cells were additionally supplemented with 2.5 µg/ml of amphotericin B (Sigma, A2942) at the time of plating, which we have previously shown[4] to increase virus titers. For VSV-G pseudotyped libraries 293T cells were used (we used cells not expressing any ACE2 in order to avoid any selection of spike, which can still be present on the surface of these VSV-G pseudotyped viruses). 12-15 hours post infection unintegrated viral genomes were recovered using QIAprep Spin Miniprep kit and prepared for Illumina sequencing as described previously[5].

For each variant functional score was calculated by getting a log enrichment ratio: $log_2\left(\left[n^{v}_{post} / n^{wt}_{post}\right] / \left[n^{v}_{pre} / n^{wt}_{pre}\right]\right)$, where n^{v}_{post} is the count of variant v in the post-selection (spike-pseudotyped) infection, n^{v}_{pre} is the count of variant v in the pre-selection (VSV-G-pseudotyped) infection, and n^{wt}_{post} and n^{wt}_{post} are the counts of variants without mutations, i.e. wildtype spike, in each condition. Positive functional scores indicate variant is able to enter cells better than wildtype and negative functional scores indicate variant is worse at entering the cells than wildtype.

The multi-dms software package[10] was used to fit a global epistasis model[82] with a sigmoid global epistasis function to the variant functional scores and to calculate mutation-level effects on cell entry. See https://dms-vep.github.io/SARS-CoV-2_XBB.1.5_spike_DMS/notebooks/func_effects_global_epistasis_Lib1-230614_high_ACE2.html for an example of this fitting for one library; the HTML documentation of the pipeline linked in the *Data availability* section has links to comparable fitting notebooks for each library.

The cell entry effects we describe in the paper are based on the cell entry experiments done on 293T-cells overexpressing high amounts of ACE2 as opposed to medium ACE2 expressing cells. Expression of more ACE2 in 293T-cells leads to higher virus titers on these cells and therefore the fitting of global epistasis model on data from these cells is slightly better.

Use of non-neutralizable standard for ACE2 binding and serum selection experiments

For both ACE2 binding and serum selection experiments a non-neutralizable standard was used in order to enable conversion of sequence counts to absolute neutralization[4]. We have previously described the use of VSV-G pseudotyped virus as the non-neutralizable standard in antibody selection experiments[4], and that VSV-G standard was also used for selections with soluble ACE2 protein to measure receptor binding since VSV-G is not neutralized by ACE2. For serum selections, we found that high concentrations of serum appreciably neutralize VSV-G itself making it not suitable as a non-neutralizable standard. We screened multiple alternative viral entry proteins and found that the RDPro glycoprotein, a modified version of an endogenous feline virus RD114 containing HIV R-peptide[83] that we further modified to contain MLV-A cytoplasmic tail to improve pseudovirus titers[84], was not neutralized even at high serum concentrations (data not

shown). The full sequence of RDPro viral entry protein used in this study can be found at https://github.com/jbloomlab/SARS-CoV-2-XBB.1.5_Spike_DMS_validations/blob/main/plasmid_maps/3737_HDM_RDPro_Twist_MLV-A_HIV-pep_correction.gb . RDPro envelope pseudotyped viruses were produced from cells with integrated barcoded lentivirus genomes as described previously for VSV-G pseudotyped standard[4]. Because RDPro-pseudotyped lentivirus titers were ~10^4 TU/ml, we further concentrated virus stocks using Lenti-X™ Concentrator (Takara, 631232) to between 3.5*10^5-1.5*10^6 TU/ml. Given that producing high titer RDPro stocks is more time consuming than making VSV-G stocks we chose to not switch to using RDPro for ACE2 binding experiments, since VSV-G is not neutralized by soluble ACE2. Note that both RDPro and VSV-G non-neutralizable standards contain the same barcodes and therefore as long as the non-neutralizable standard is not neutralized, the results should remain the same regardless of the standard used.

Recombinant Protein Production

SARS-CoV-2 spike ectodomain and human ACE2 ectodomains were expressed and purified as described previously[18,19] Mutant Spike ectodomain constructs were designed in the BA.2 and XBB.1.5 backgrounds with HexaPro[26] mutations, N terminal "MFVFLVLLPLVSS" signal peptide, C terminal GSSG linker, foldon, linker, avi-8x polyhistidine tag, and were cloned into a pCDNA3.1(+) vector. A222M, N405A, A570F, A570D, A701M, D950N, R493Q, R498V mutations were evaluated in the BA.2 background; Q115K, T167I, N234T, N405A, Q762L, F1121L, R498V, Q804L, Q493L, G614D, F371N, A222M mutations were evaluated in XBB.1.5 background. Expi293F cells were diluted to a density of 3 million cells per mL and transfected using ExpiFectamine 293 Transfection Kit (Thermo Fisher Scientific). Cells were incubated shaking at 130 rpm at 37°C and 8% CO_2. Three to four days post transfection proteins were purified from clarified supernatants. Human ACE2 ectodomains were purified using 1mL Histrap Fast Flow nickel affinity columns (Cytiva), and washed with 20 mM imidazole, 25 mM sodium phosphate pH 8.0, and 300 mM NaCl prior to elution with an imidazole gradient using a buffer containing 500 mM imidazole pH 8.0, 25 mM sodium phosphate, 300 mM NaCl pH 8.0. SARS-CoV-2 spike ectodomains were purified using 1mL of Ni Excel resin (Cytiva) and washed with 40 mM imidazole pH 8.0, 25 mM sodium phosphate pH 8.0, and 300 mM NaCl prior to elution with 300 mM imidazole pH 8.0, 25 mM sodium phosphate pH 8.0, and 300 mM NaCl. SARS-CoV-2 spike ectodomains were buffer exchanged into 20 mM sodium phosphate pH 8.0, and 100 mM NaCl (PBS) using centrifugal filters (corning) with a MWCO of 100 kDa. Purified BA.2 and XBB.1.5 S variants were analyzed by negative stain electron microscopy to confirm retention of proper folding and monodispersity (**Extended Data Fig. 3**). Human ACE2 ectodomain were concentrated using centrifugal filters (Corning) with a MWCO of 30kDa and were further purified by size exclusion chromatography and run through a Superdex 200 Increase 10/300 GL column (Cytiva) pre-equilibrated in PBS. All proteins were analyzed by SDS-PAGE for purity, then flash frozen and stored at -80°C. For deep mutational scanning ACE2 binding experiments biotinylated dimeric ACE2 was purchased from ACROBiosystems (AC2-H82E7-1mg).

ACE2 binding measurement using deep mutational scanning libraries

Previous research has shown that soluble ACE2 can neutralize SARS-CoV-2 variants with potency proportional to virus binding to the receptor[1,16]. We used this observation to measure the effects of mutations in our deep mutational scanning libraries on ACE2 binding.

As described previously[4], before starting ACE2 binding experiments we spiked-in a VSV-G non-neutralizable standard at 1-2% of the total virus titers used. ~1 million virus transcription units per sample were incubated with soluble monomeric or dimeric ACE2 at 37˚C for 1 h before being added to 293T-ACE2 cells. 293T-ACE2 cells expressing a medium amount of ACE2 ('medium-ACE2' cells described in Farrell et al. 2021[32]) were used for all ACE2 binding experiments. For these experiments we targeted a range of ACE2 concentrations to use that would span fro

m less than IC50 to full virus neutralization in order to capture both mutations that increase ACE2 binding (those that are neutralized by soluble ACE2 very potently) and those that decrease it (which would be more difficult to neutralize with soluble ACE2 neutralized). For monomeric ACE2 the starting concentration was 2.88 μg/ml and it was increased 3-fold for the other dilutions. For dimeric ACE2 starting concentration was 0.21 μg/ml and it was similarly increased 3-fold for the other dilutions. 12-15 hours post infection non-integrated lentiviral genomes were extracted from cells and

barcode sequencing libraries were prepared as described previously[4]. ACE2 binding experiments were performed with two biological replicates for each library.

Analysis of mutation-level effects and fitting of neutralization curves to the data was performed using *polyclonal* software[82] version 6.9. Examples of polyclonal model fitting for monomeric ACE2 data can be found at:

- For XBB.1.5 full spike libraries https://dms-vep.github.io/SARS-CoV-2_XBB.1.5_spike_DMS/notebooks/fit_escape_ACE2_binding_Lib1-230614-monomeric_ACE2.html
- For XBB.1.5 RBD only libraries https://dms-vep.github.io/SARS-CoV-2_XBB.1.5_RBD_DMS/notebooks/fit_escape_ACE2_binding_Lib1-230615-monomeric-ACE2.html
- For BA.2 libraries https://dms-vep.github.io/SARS-CoV-2_Omicron_BA.2_spike_ACE2_binding/notebooks/fit_escape_ACE2_binding_Lib1-230114-monomeric_ACE2.html

The HTML documentation of the pipeline linked in the *Data availability* section has links to comparable fitting notebooks for each replicate library, as well as dimeric ACE2 selection data available for XBB.1.5 RBD only libraries.

Mass photometry

Mass photometry was performed on a Refeyn TwoMP system (Refeyn Ltd). Microscope cover slides were rinsed with isopropanol and Milli-Q water then dried under nitrogen flow. Sample chambers were assembled using silicon gaskets, and the instrument lens coated with immersol before placing slides on the MP sample stage. Samples were added to the sample chamber and the instrument was focused immediately prior to each data acquisition. Spike ectodomain samples were diluted to 25 nM for all data acquisitions. Spike was mixed with 0, 25, 50, 75 or 100 nM of dimeric human ACE2 ectodomain and incubated at room temperature for 5 min prior to data acquisition. MP image data was analyzed in Refeyn DiscoverMP, using in-house protein standards for mass calibration, and processed single-particle mass detection events were exported for determination of RBD-ACE2 occupancy. The mass events were truncated to a range of 0-1600 kDa for apo spike runs and to 350-1300 kDa for spike-ACE2 runs, thereby excluding multiple spikes cross-linked by one or multiple ACE2 dimers, as well as free ACE2 (which is not used to calculate RBD occupancy). Retained mass events for each run were used to estimate two-, three-, or four-component Gaussian mixture models (GMMs) with Scikit-Learn[83], and each component was assigned as representing unbound spike, or 1 ACE2-, 2 ACE2-, or 3 ACE2-bound spike trimers if its molecular mass (Gaussian mean) fell between 400-600 kDa, 600-800 kDa, 800-1000 kDa, or 1000-1200 kDa, respectively. The relative abundance of each of the four species, and thus RBD-ACE2 occupancy, were determined from the respective weights (proportions of overall probability mass) of the Gaussian components, as follows: $RBD\ occupancy = \frac{S^{1xACE2}+2S^{2xACE2}+3S^{3xACE2}}{S^{total}\times 3}$, $S^{1xRBD}\ occupancy = \frac{S^{1xACE2}+S^{3xACE2}}{S^{total}}$, $S^{2xRBD}\ occupancy = \frac{S^{2xACE2}+}{S^{total}}$, $S^{3xRBD}\ occupancy = \frac{S^{3xACE2}}{S^{total}}$ where S^{1xACE2} is the weight of the respective Gaussian components for single ACE2 bound spike, S^{2xACE2} is Gaussian component spike bound by 2 ACE2 molecules, S^{3xACE2} is Gaussian component spike bound by 3 ACE2 molecules and S^{total} is the sum of Gaussian components for spike bound by no, one, two or three ACE2 molecules (**Extended Data Fig. 4a**). For visualization of the modeling results (**Supplementary Figure 2-3. 3-4**) and for selection of the number of Gaussian components appropriate for each sample, each GMM was used to predict a (continuous) mass frequency distribution, which was area-scaled and overlaid on the corresponding full-range mass event histogram.

Serum escape mapping using deep mutational scanning libraries

Before use, sera were heat inactivated at 56°C for 1 hour to eliminate complement activity. XBB* infection sera neutralization was determined using standard pseudovirus neutralization assay described in Crawford et al. (2020)[5]. The sequence of the spike expression plasmid used for these experiments is provided at https://github.com/jbloomlab/SARS-CoV-2-XBB.1.5_Spike_DMS_validations/blob/main/plasmid_maps/HDM_XBB15.gb . Using these measurements we determined the amount of serum needed to neutralize the virus at IC98-IC99. As described previously[4], before starting selections we spiked-in a non-neutralizable standard at 1-2% of the total virus titers

used. RDPro pseudotyped non-neutralizable standard was used for all serum selections to avoid non-specific standard neutralization (see section *Use of non-neutralizable for ACE2 and serum selection experiments* above). For sera selection experiments ~1 million transcription units for each library sample were used. Libraries were incubated at three increasing serum concentrations starting with IC98-IC99 (depending on serum volume available) and increasing serum concentration 4-fold at each dilution. These serum concentrations were selected so that only a small percentage of variants would be able to pass sera selection, therefore selecting for strongest escape variants. Additional sera concentrations are used to cover a greater dynamic range as sometimes neutralization values determined against wild-type spike using luciferase-based system do not quite match neutralization values for library virus pool. Virus-serum mixtures were incubated for 1 h at 37˚C and subsequently 293T-ACE2 cells were infected with them. We used medium ACE2 expressing cells for all serum selection experiments ('medium-ACE2' cells in Farrell et al. 2021[32]) although as shown in **Extended Data Fig. 5** we did not detect major differences in serum escape compared to cells with high ACE2 expression. 12-15 hours post infections non-integrated lentiviral genomes were extracted from cells and barcode sequencing libraries were prepared as described previously[4].

Polyclonal software[82] (version 6.9) was used to analyze mutation-level escape and fit neutralization curves to the data. An example for data fitting for one sera sample can be found at https://dms-vep.github.io/SARS-CoV-2_XBB.1.5_spike_DMS/notebooks/fit_escape_antibody_escape_Lib1-230815-sera-343C_mediumACE2.html for XBB.1.5 full spike library and at https://dms-vep.github.io/SARS-CoV-2_XBB.1.5_RBD_DMS/notebooks/fit_escape_antibody_escape_Lib1-230815-sera-343C_mediumACE2.html for XBB.1.5 RBD library. The HTML documentation of the pipeline linked in the *Data availability* section has links to comparable fitting notebooks for each biological replicate, as well as all other sera fits.

Validation of escape using standard pseudovirus neutralization assay

To validate serum escape mutations, we cloned desired point mutants into an expression plasmid coding for XBB.1.5 spike. The sequence of this XBB.1.5 expression plasmid is at https://github.com/jbloomlab/SARS-CoV-2-XBB.1.5_Spike_DMS_validations/blob/main/plasmid_maps/3813_HDM_XBB15_with_F490S.gb (note this spike sequence contains F490S mutation). Pseudoviruses were generated and titrated as described in Crawford et al. (2020)[5] except that pHAGE6_Luciferase_IRES_ZsGreen backbone was used which requires only Gag/Pol (BEI: NR-52517) helper plasmid to produce pseudoviruses. Pseudovirus stocks were diluted to stock concentration of >200,000 relative light units per ul and neutralization assays were performed on medium-ACE2 cells. Starting serum dilution for neutralization assays was 1:30 and it was serially diluted 1:3 to generate neutralization curves. Neutralization curves were plotted by fitting a Hill curve to fraction infectivity data for each variant. This was done using *neutcurve* package (https://jbloomlab.github.io/neutcurve/, version 0.5.7). Analysis notebook for neutralization curves is at https://github.com/jbloomlab/SARS-CoV-2-XBB.1.5_Spike_DMS_validations/tree/main.

Cells

All cells in this study were maintained in D10 media (Dulbecco's Modified Eagle Medium with 10% heat-inactivated fetal bovine serum, 2 mM l-glutamine, 100 U/mL penicillin, and 100 μg/mL streptomycin). 293T-ACE2 cells expressing medium amount of ACE2 ('medium-ACE2' cells described in Farrell et al. 2021[32]) were additionally supplemented to 2 μg/ml of doxycycline. Cells used to store spike libraries were maintained in media supplemented with doxycycline-free FBS as described previously[4].

Ethics statement

XBB* infection sera were collected after informed consent from participants in the prospective longitudinal Hospitalized or Ambulatory Adults with Respiratory Viral Infections (HAARVI) study from Washington State, USA, which was approved by University of Washington Institutional Review Board (protocol #STUDY00000959).

Comparison of deep mutational scanning phenotypes to changes in clade growth

To estimate clade growth rates, we used a multinomial logistic regression model of global lineage frequency data. GISAID sequences were obtained from the bulk .fasta download (dated 2023-10-02) and processed with Nextclade (v2.14.0)

using the BA.2 reference (`sars-cov-2-21L`). Using Nextclade quality metrics, only sequences with >90% coverage and an overall QC status of "good" were retained. Since outlier dates could distort model estimates, we required sequences to have a fully specified deposition and collection date (ie. year, month, and day), and to have a collection date within 150 days of deposition (primarily to avoid collection dates where the year was incorrectly annotated). Finally, for each lineage, we excluded sequences with dates that were extreme outliers for that specific lineage, falling outside of 3.5 times the interquartile range of the median. For the model fit, we retained countries with >500 sequences, and lineages with >200 sequences. Counts for each lineage were aggregated per country, per day.

We model lineage counts using multinomial logistic regression. For the ith lineage, g_i denotes the global (ie. shared across all regions) per-lineage relative growth rate parameter (these are the parameters we wish to estimate), and c_{ij} is a "nuisance" intercept parameter for the ith lineage in the jth region. We assume that the ratio of the frequency of any two lineages varies approximately exponentially over time *t* (here in years), and model the probability of the nth sample $S^t_j[n]$ being from lineage *i* as:

$$P(S^t_j[n] = i) = \frac{e^{tg_i + c_{ij}}}{\Sigma_i e^{tg_i + c_{ij}}}$$

We use gradient descent to minimize the negative log probability $-\Sigma_n \log[P(S^t_j[n] = i)]$, with the relative growth rate parameters "centered" to have their mean equal to zero, removing a superfluous degree of freedom. Growth rates are interpreted such that the ratio of the frequencies of two lineages changes with time as

$$\frac{P(S^t_j[n]=i)}{P(S^t_j[n]=q)} = K \cdot e^{t(g_i - g_q)}$$

where g_i - g_q is the difference in the growth rates between the two lineages and, for a given region, $K=\exp(c_{ij}-c_{qj})$ is a constant determined by their intercepts.

The model was implemented in the Julia language, using Flux.jl and CUDA.jl to allow for GPU computation, and optimized using Flux's "AdamW" optimizer with very weak (10^{-10}) "weight decay" numerical regularization of the parameters. The model implementation can be viewed at: https://github.com/MurrellGroup/MultinomialLogisticGrowth.

Visual inspection of the model fits to count data, aggregated daily for each lineage and region, showed an acceptable fit at the region level, despite using globally shared growth rate parameters, indicating relatively consistent growth rates across regions. A representative example (from Switzerland) is shown at **Supplementary Figure 5**. See https://github.com/MurrellGroup/MultinomialLogisticGrowth/tree/main/plots for similar visualizations across all regions used in the global model fit. Note that when a region has no samples for a given period, this will not inform the growth rate estimates, but we still plot the model's expected lineage frequencies.

The growth rate estimates themselves are at https://github.com/MurrellGroup/MultinomialLogisticGrowth/blob/main/model_fits/rates.csv. For the analyses in this paper, we considered only growth estimates from XBB descended clades with at least 400 sequences, since clades with more sequences have more accurate growth estimates. The definitions of the clades (e.g., which mutations they contain) as well as their phylogeny (parents and descendants for each clade) were taken from https://github.com/corneliusroemer/pango-sequences/blob/main/data/pango-consensus-sequences_summary.json.

As described in the results and **Fig. 6** and **Extended Data Figs. 10** and **11**, directly predicting growth rates of clades from the deep mutational scanning is a confounded approach due to both phylogeny and the simple fact that newer clades tend to have both more spike mutations and higher growth rates, leading to a trivial correlation of clade growth rate with number of spike mutations. The real question of interest is not whether more fit clades with additional mutations will be selected over time (we know they will), but rather which of the mutant clades present at any given time will be more successful. Therefore, as indicated in **Extended Data Fig. 10b**, we computed the *change* in growth rate for each parent-descendant clade pair with estimates for both clade members and at least one spike mutation. We then also computed the change in each spike phenotype as measured by deep mutational scanning for the clade pairs based on the mutations separating the pair members, simply adding together the mutation effects for pairs separated by multiple spike mutations. Non-spike mutations were ignored. The Pearson correlations with each phenotype are shown in **Fig. 6a**, and the statistical significance of the correlations were assessed by randomizing the deep mutational scanning measurements among mutations 100 times and assessing how many randomizations had a correlation greater than or equal to the observed value. To test the predictive value of combining all spike phenotypes, we performed a similar

analysis but using ordinary least squares multiple linear regression on all three phenotypes. Those results are shown in **Fig. 6b**, with the significance again assessed by comparing the actual Pearson correlation to that generated by fitting the model to data randomized among sites. To compute the unique variance explained by each phenotype, we removed the phenotypes one-by-one and computed the unique variance explained as the squared Pearson correlation for the full model minus the squared Pearson correlation for the model with that phenotype removed. See https://dms-vep.github.io/SARS-CoV-2_XBB.1.5_spike_DMS/notebooks/current_dms_compare_natural.html for the notebook performing this analysis, and https://github.com/dms-vep/SARS-CoV-2_XBB.1.5_spike_DMS/blob/main/results/compare_natural/current_dms_clade_pair_growth.csv for the numerical data on the clade pairs and their changes in spike phenotypes.

We compared the predictive value of the full-spike deep mutational scanning to the predictive value of models based on several other values. The first such comparator model simply involves counting the change in number of spike mutations relative to Wuhan-Hu-1 in each clade pair; as shown in **Extended Data Fig. 10f**, this model has no predictive value.

The second comparator model uses the effect of RBD mutations on ACE2 affinity and RBD expression as measured in yeast-display deep mutational scanning of the XBB.1.5 RBD[20] as well as per-site escape values (same value assigned to each mutation at each site) as computed using the default settings of the antibody escape calculator[8] at https://github.com/jbloomlab/SARS2-RBD-escape-calc/tree/5ebb88e5b8c9adc1b601b3cb1cc5308532d97a38 which is based on monoclonal antibody deep mutational scanning data[84]. For this model, all non-RBD mutations were assigned a value of zero for all phenotypes. As shown in **Extended Data Fig. 11b,d**, the predictions of this model are not significant at a level of P = 0.05 compared to models with the measurements randomized among sites.

The third comparator model uses the effects of mutations to XBB.1.5 as estimated using the EVEscape method (https://evescape.org/data)[49]. As shown in **Extended Data Fig. 11c**, the predictions of this model are not significant at a level of P = 0.05.

The numerical data used for all the comparator models is at https://github.com/dms-vep/SARS-CoV-2_XBB.1.5_spike_DMS/tree/main/data/compare_natural_datasets.

Serum sample	Sex	Race	Age	Number of infections	Number of vaccine and booster doses	1st infection symptom date	2nd infection symptom date	3rd infection symptom date	Vaccine dose 1 date	Vaccine dose 2 date	Vaccine dose 3 date	Vaccine dose 4 date	Vaccine dose 5 date	Vaccine dose 6 date
287C	Female	White	57	3	5	Sep-21	May-22	Mar-23	Dec-20	Jan-21	Dec-21	Jun-22	Nov-22	
288C	Male	White	56	3	5	Aug-21	May-22	Mar-23	Jan-21	Feb-21	Dec-21	Jun-22	Nov-22	
343C	Female	Asian	19	2	4	Jan-22	Apr-23		Mar-21	Apr-21	Dec-21	Sep-22		
493C*	Male	White	28	1	2	Jan-23			Jan-21	Feb-21				
497C	Female	White	48	2	4	Apr-22	Mar-23		Dec-20	Jan-21	Sep-21	Oct-22		
498C*	Male	White	70	1	5	Dec-22			Jan-21	Jan-21	Nov-21	May-22	Oct-22	
500C	Male	Asian	63	1	5	Mar-23			Apr-21	Apr-21	Nov-21	Apr-22	Sep-22	
501C	Male	White	53	1	5	Mar-23			Mar-21	Mar-21	Sep-21	Apr-22	Oct-22	
503C	Male	White	51	1	6	May-23			Apr-21	Apr-21	Nov-21	Apr-22	Sep-22	Mar-23
505C*	Female	Middle Eastern	41	1	3	Feb-23			Apr-21	May-21	Dec-21			
* sequencing confirmed infection. 493C and 505C had XBB.1.5 infection and 498C had XBB infection.														

Supplementary table 1: Information on sera used in this study

Sera used in this study. Table shows the number and dates for infections and vaccinations each individual had. All individuals either had a confirmed XBB* infection (marked by * in the table above) or had the last recorded infection during the period when XBB or its descendant lineages were the most common circulating variants in Washington state. 493C and 505C had XBB.1.5 infection and 498C had XBB infection. In February 2023 70% of sequenced cased were confirmed XBB or its descendant lineages and between March and May this number grew from 88% to 97% according to the samples sequenced at University of Washington Virology labs[82].

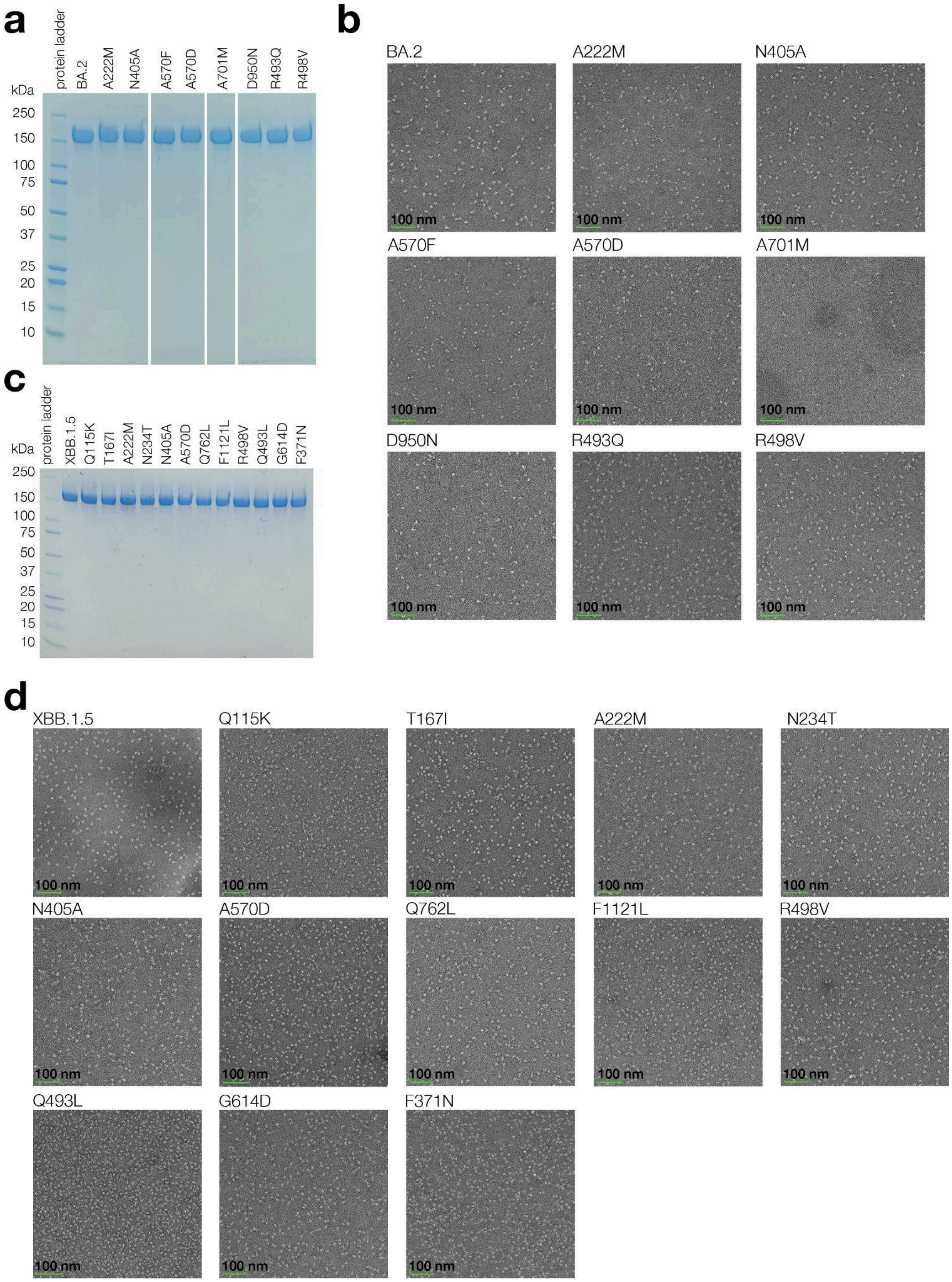

Supplementary figure 1: BA.2 and its mutant spike preparations

a, Reducing SDS-PAGE gel for purified BA.2 wildtype and mutant spike ectodomains. All constructs are pre-fusion stabilized with HexaPro mutations[26]. 3µg of purified protein loaded. Single

major band for all samples confirms sample purity. **b,** Negative stain electron microscopy images for the purified BA.2 spike mutants to confirm proper folding and monodispersity of the samples. **c,** Reducing SDS-PAGE gel for purified XBB.1.5 wildtype and mutant spike ectodomains. All constructs are pre-fusion stabilized with HexaPro mutations[26]. **d,** Negative stain electron microscopy images for the purified XBB.1.5 spike mutants. Two independent protein preparations were made for each spike variant, plots a-d show representative gels and micrograps for one of the protein preparations.

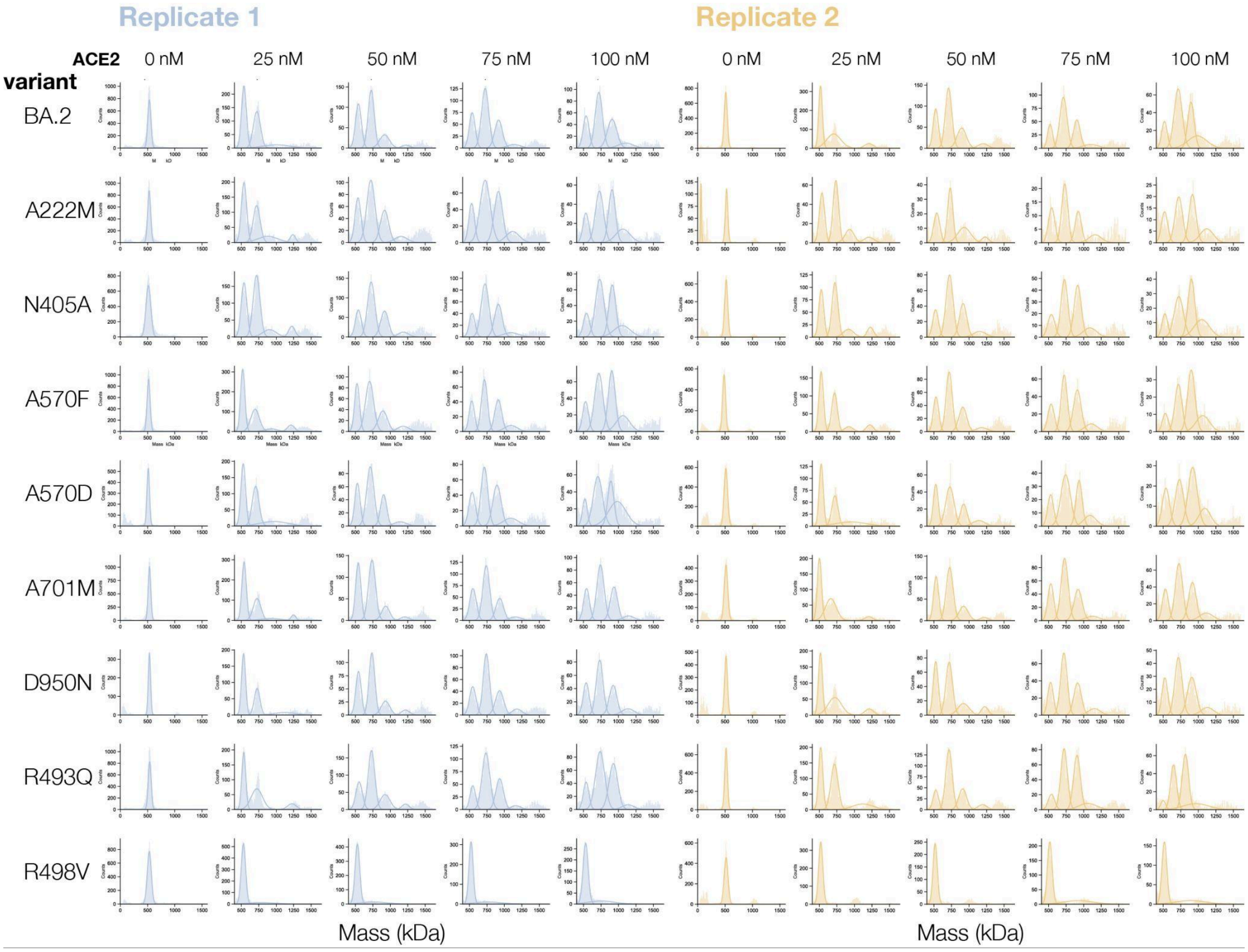

Supplementary figure 2: Mass photometry measurements for individual BA.2 spike variants

Spike molecular mass distributions measured using mass photometry for each biological replicate (blue and orange) corresponding to independent purification batches. Each row shows a BA.2 spike mutant and each column shows measurements at different ACE2 concentrations. In the absence of ACE2, some samples had a small peak to the left which may be a misfolded spike monomer[70] which was present only in some protein preparations and is excluded from Gaussian curve fitting in the presence of ACE2.

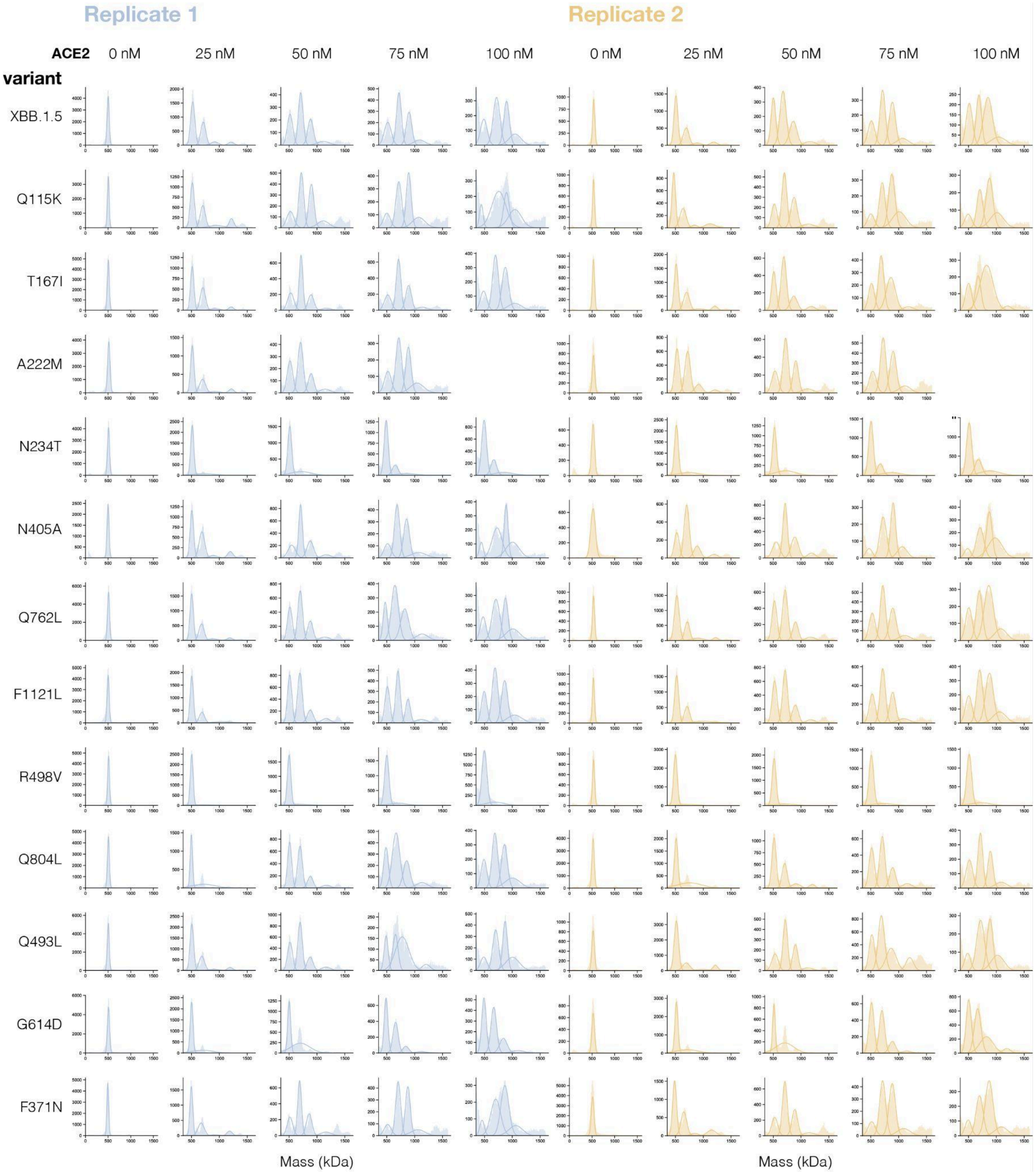

Supplementary figure 3: Mass photometry measurements for individual XBB.1.5 spike variants

Spike molecular mass distributions measured using mass photometry for each biological replicate (blue and orange) corresponding to independent purification batches. Each row shows an XBB.1.5 spike mutant and each column shows measurements at different ACE2 concentrations. In the absence of ACE2, some samples had a small peak to the left which may be a misfolded spike

monomer[70] which was present only in some protein preparations and is excluded from Gaussian curve fitting in the presence of ACE2.

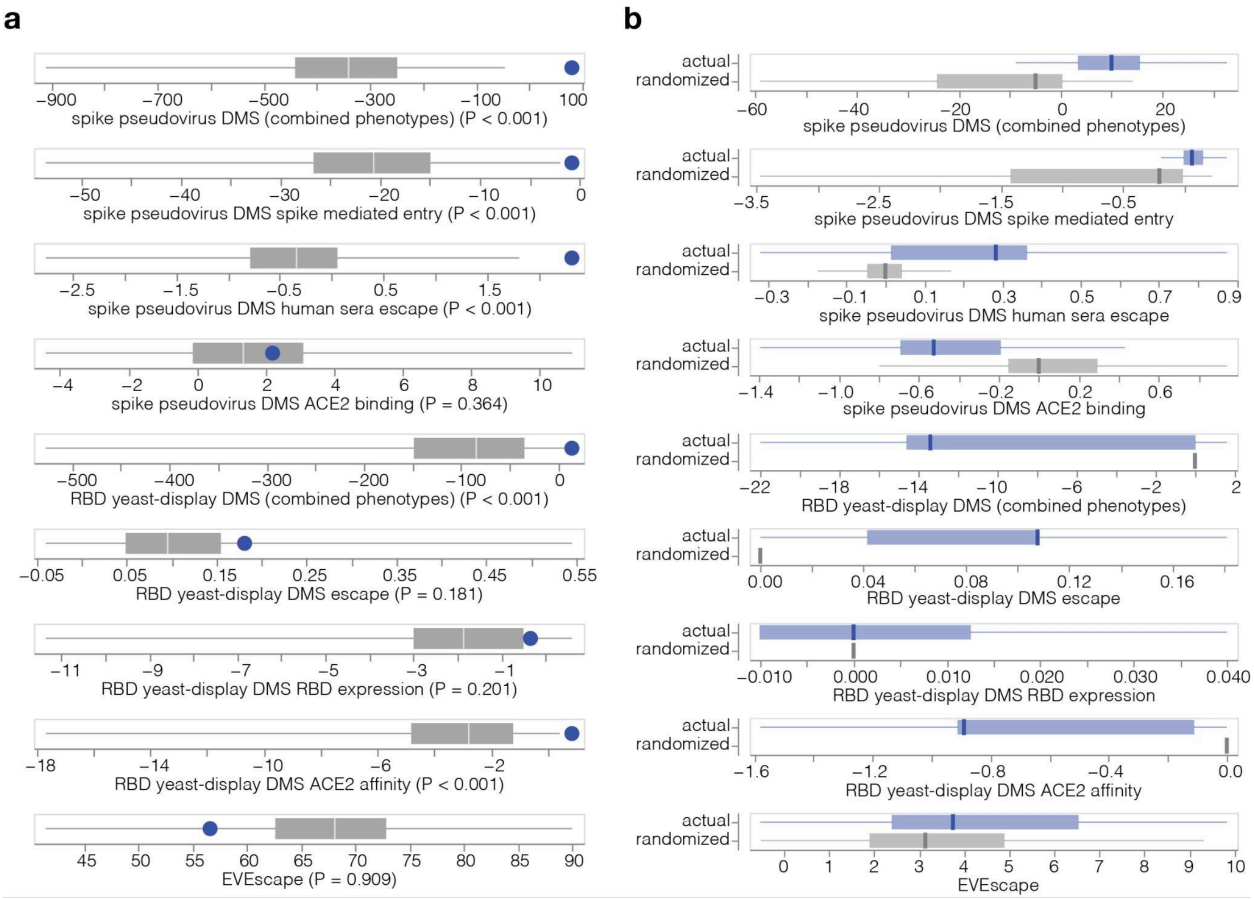

Supplementary figure 4: Ability of various spike properties to distinguish the actual BA.2.86 and BA.2.86-descended clades from randomly mutated sequences

This figure assesses the ability of various spike properties to correctly identify that BA.2.86 and its descendant clades (which have spread widely and so by definition have high fitness) from other sequences with the same number of mutations drawn randomly from mutations observed in human SARS-CoV-2 sequences. This test is inspired by that used in the ninth extended data figure of Thadani et al[49]. a, The blue circles show the phenotype of the actual BA.2.86 spike relative to its parent BA.2 as computed from the sum of the mutation effects measured in the XBB.1.5 full-spike deep mutational scanning, XBB.1.5 RBD yeast-display, or predicted by EVEscape. The grow shows min-max boxplots the phenotypes of n=1,000 spike sequences generated by adding to the BA.2 spike the same number of amino-acid mutations in BA.2.86 relative to BA.2, drawing the mutations randomly from the set of all amino-acid mutations observed at least 50 times in GISAID. The P-value represents the fraction of these randomly mutated sequences with phenotypes at least as favorable as that of the actual BA.2.86 spike. Therefore, when the blue circle is far to the right of the gray distribution (small P value) that means the spike phenotype is highly effective at distinguishing the actual high-fitness BA.2.86 spike from randomly mutated sequences. For the panels labeled “spike pseudovirus DMS (combined phenotypes)” and “RBD yeast-display DMS (combined phenotypes)”, the phenotype is a linear combination of the three phenotypes measured

in each type of deep mutational scanning weighted with the coefficients determined by the multiple-linear regression on XBB-descended clades (see Fig. 6b and Extended Data Fig. 11). Overall, this figure shows that full-spike pseudovirus sera escape, full-spike pseudovirus cell entry, and RBD yeast-display ACE2 affinity are the three phenotypes with the best ability to distinguish the actual BA.2.86 spike from randomly mutated sequences. P-values are for one-sided tests of the hypothesis that the tested predictor outperforms randomizations, and are reported individually for each comparison. **b**, An analysis conceptually similar to that in panel a but comparing all designated Pango clades descended from BA.2.86 to their parental BA.2.86 versus a set of n = 100 randomly mutated sequences generated by adding the same number of random mutations (observed at least 50 times in GISAID). The blue min-max boxplots show the distribution of the phenotype among the actual BA.2.86-descended clades that have evolved, whereas the gray shows the distribution of the phenotype among the randomly mutated sequences. When the blue distribution is shifted far to the right relative to the gray distribution, that indicates that the phenotype can effectively distinguish the actual clades that have evolved from randomly mutated ones. Whiskers in a-b extend to minimum and maximum data points and vertical lines indicate median. See https://dms-vep.org/SARS-CoV-2_XBB.1.5_spike_DMS/notebooks/compare_BA.2.86.html for the computer code implementing the analysis shown here.

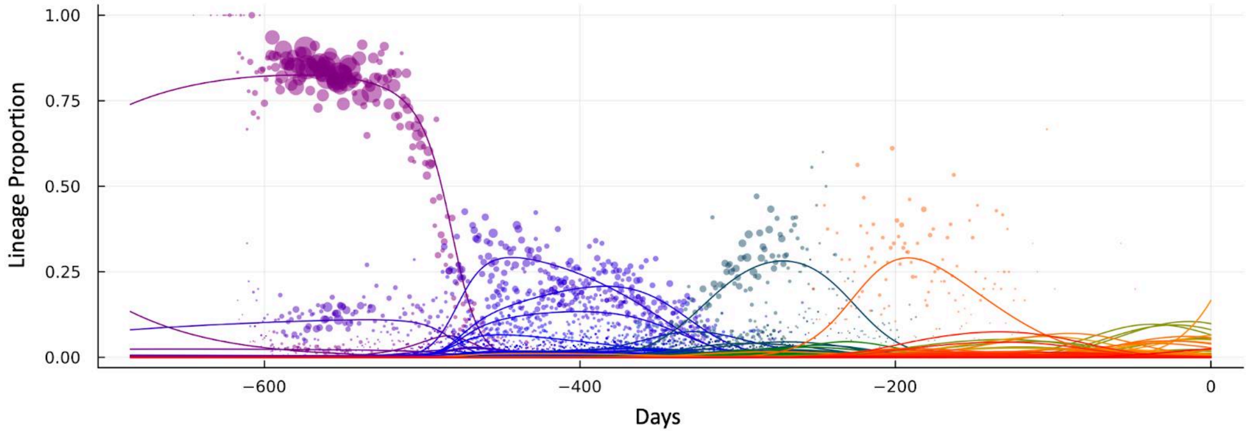

Supplementary figure 5: Example of model fit to lineage counts

A representative example of the modeled lineage counts using multinomial logistic regression (lines) versus actual lineage counts (points) for Switzerland. Each color represents a different clade. These fits were used to estimate the clade growth rates.

Supplementary references

76. Khare, S. *et al.* GISAID's role in pandemic response. *China CDC Wkly.* **3**, 1049 (2021).

77. Turakhia, Y. *et al.* Ultrafast Sample placement on Existing tRees (UShER) enables real-time phylogenetics for the SARS-CoV-2 pandemic. *Nat. Genet.* **53**, 809–816 (2021).

78. Bloom, J. D. An Experimentally Determined Evolutionary Model Dramatically Improves Phylogenetic Fit. *Mol. Biol. Evol.* **31**, 1956–1978 (2014).

79. Otwinowski, J., McCandlish, D. M. & Plotkin, J. B. Inferring the shape of global epistasis. *Proc. Natl. Acad. Sci.* **115**, E7550–E7558 (2018).

80. Bell, A. J., Fegen, D., Ward, M. & Bank, A. RD114 envelope proteins provide an effective and versatile approach to pseudotype lentiviral vectors. *Exp. Biol. Med.* **235**, 1269–1276 (2010).

81. Tomás, H. A. *et al.* Improved GaLV-TR Glycoproteins to Pseudotype Lentiviral Vectors: Impact of Viral Protease Activity in the Production of LV Pseudotypes. *Mol. Ther. - Methods Clin. Dev.* **15**, 1–8 (2019).

82. Yu, T. C. *et al.* A biophysical model of viral escape from polyclonal antibodies. *Virus Evol.* **8**, veac110 (2022).

83. Pedregosa, F. *et al.* Scikit-learn: Machine Learning in Python. *J. Mach. Learn. Res.* **12**, 2825–2830 (2011).

84. Yisimayi, A. *et al.* Repeated Omicron exposures override ancestral SARS-CoV-2 immune imprinting. *Nature* **625**, 148–156 (2024).

85. UW Virology COVID-19 Dashboard. https://depts.washington.edu/labmed/covid19/#sequencing-information.
